# Investment in reward by ant-dispersed plants consistently selects for better partners along a geographic gradient

**DOI:** 10.1093/aobpla/plz027

**Published:** 2019-05-02

**Authors:** Nataly Levine, Gilad Ben-Zvi, Merav Seifan, Itamar Giladi

**Affiliations:** Mitrani Department of Desert Ecology, Swiss Institute for Dryland Environmental and Energy Research, Jacob Blaustein Institutes for Desert Research, Ben-Gurion University of the Negev, Sede Boqer Campus, Midreshet Ben-Gurion, Israel

**Keywords:** Elaiosome, Mediterranean ecosystem, mutualism, myrmecochory, partner choice hypothesis, seed dispersal, *Sternbergia clusiana*

## Abstract

Seed dispersal by ants (myrmecochory) is an asymmetric, presumably mutualistic interaction, where a few ant species benefit many plants. Myrmecochorous plants express specialized adaptations, most notably a large elaiosome, which promote interactions with efficient seed dispersers while decreasing interactions with poor dispersers, resulting in *de facto* partner choice. However, because variation in plants’ investment in reward and ant response to them may vary spatially and temporally, it is unclear whether such specialization is consistent along geographic gradients; especially towards myrmecochory’s range margin. To answer this question on context-dependent partner choice, we first estimated variation in reward investment by co-occurring myrmecochores along a steep environmental gradient in a Mediterranean region. Second, we tested whether variation in plant investment in reward was positively and consistently correlated with the quality of dispersal plant received along the same gradient. Using *in situ* cafeteria experiments, we simultaneously presented diaspores of locally co-occurring myrmecochorous species to ants of two guilds representing high- and low-quality dispersers. We then recorded ant-seed behaviour, seed preference and seed removal rates for each ant guild. We found both overall and within-site high variation among plant species in the total and relative investment in elaiosomes. Both ant guilds removed substantial proportion of the seeds. However, scavenging ants (high-quality dispersers) clearly preferred diaspores with larger elaiosomes, whereas granivorous ants (low-quality dispersers) exhibited no preference. Furthermore, both the variation in plant traits and the corresponding response of different ant guilds were consistent along the studied geographic gradient. This consistency holds even when granivores, which removed seeds in a non-selective fashion and provided apparently low-quality seed dispersal services, were, at least numerically, the dominant ant guild. This dominance and the consistency of the partner choice shed light on the functionality of elaiosomes at the margins of myrmecochory’s distribution.

## Teaser

Seed dispersal by ants is a mutualistic interaction in which some ants disperse the seeds of plants, which produce a lipid-rich seed appendage that serves as a reward to the ants. The strength of this interaction varies with the quality of the reward, by the quality of dispersal provided by the ants and by the environmental context.

We found that, among co-occurring plant species, those that produced larger rewards were preferred by high-, but not by low-quality seed-dispersing ant species. This preference pattern was consistent along a sharp geographic gradient in a Mediterranean ecosystem.

## Introduction

Seed dispersal by animals is often portrayed as a network of mutualistic interactions, involving many plant and animal species that exchange resource (e.g. fleshy fruit) for a service (dispersal). Although such networks are often diffuse, involving many interacting species (Bascompte and Jordano 2007), they are also asymmetric and heterogeneous with high among-species variation in both the interaction frequency and the per-interaction effect (Hoeksema and Bruna 2000; Lengyel *et al.* 2009, 2010). An important seed dispersal interaction that has been recently recognized as an asymmetric, functionally specific and context- and partner-dependent interaction is myrmecochory, seed dispersal by ants (Gove *et al.* 2007; Manzaneda and Rey 2009; Ness *et al.* 2009; Warren and Giladi 2014; Warren *et al.* 2019).

Myrmecochory has a worldwide, yet uneven, distribution with ~11 000 plant species exhibiting adaptation for dispersal by ants, and ~100 ant species that are considered as effective seed dispersers (Warren and Giladi 2014). The defining feature of myrmecochorous plants is the elaiosome, a lipid-rich seed appendage that attracts certain groups of ant species and elicits seed carrying behaviour (Gorb and Gorb 2003). Myrmecochorous plants consist up to 40 % of the herbaceous flora in some regions that are considered as myrmecochory’s hotspots, such as eastern North America, South-African fynbos and Australia. Although, in Australia, myrmecochores are well represented in arid zones, in the Palaearctic biogeographical region, this dispersal syndrome is common in mesic habitats and becomes rare towards drier climates (Lengyel *et al.* 2009, 2010). Across their range, myrmecochorous plants interact with a variety of seed-collecting ants that differ in their diet preferences, activity time, foraging mode, nest location and nest dynamics (Giladi 2006; Bas *et al.* 2007; Ness *et al.* 2009), which results in differences in the efficiency of seed dispersal service they provide (Hughes and Westoby 1990; Boulay *et al.* 2007*b*).

By and large, there are two distinctive guilds of seed-collecting ants. Granivorous ants are predominately seed predators that collect and consume seeds of many plant species, regardless of the presence of elaiosome. Yet, granivorous ants may contribute to effective dispersal by unintentionally dropping undamaged seeds in suitable microhabitats while foraging, and/or by leaving intact seeds in their shallow nest chambers and refuse piles (Espadaler and Gomez 1996; Detrain and Tasse 2000). The second group of seed-collecting ants includes mainly scavenging ants. Even though their main diet consists of dead insects, scavenging ants avidly collect elaiosome-bearing seeds, probably lured by the chemical composition of elaiosomes that serves as a chemical mimicry of the ants’ insect prey (Hughes *et al.* 1994; Pfeiffer *et al.* 2010). These scavenging ants forage solitarily, and after transporting the whole diaspore (seed + elaiosome) to their nest, they consume the elaiosome and discard the unharmed seed outside the nest.

It has been shown that scavenging ants provide a better dispersal service to myrmecochores than other ant guilds (Gove *et al.* 2007; Ness *et al.* 2009; Warren and Giladi 2014). Furthermore, the variation in dispersal-related plant and diaspore traits at both the interspecific level (Hughes and Westoby 1992) and the intraspecific level (Mark and Olesen 1996) may also be associated with high interaction frequency between seeds and the more beneficial ant guild. For example, high elaiosome/diaspore mass ratio serves as an attraction for larger ant species, which are capable of carrying larger seeds at higher removal rates and over larger distances (Ness *et al.* 2004; Takahashi and Itino 2012). High seed removal rate by more specialized ants decreases seed predation (Ohara and Higashi 1987; Hughes and Westoby 1990; Ohkawara *et al.* 1996; Ohkawara *et al.* 1997) and generally improves plant fitness (Hanzawa *et al.* 1988; Boulay *et al.* 2007*b*). Such observations suggest that selection has been acting on plant traits that increase the overall rate of seed dispersal and the probability of seeds being dispersed by more effective seed dispersers, resulting in *de facto* partner choice (Giladi 2006; Warren and Giladi 2014).

In this study, we test whether partner choice in myrmecochory is consistent across a geographic gradient, which is mainly associated with a sharp gradient in precipitation (Table 1). Our study was conducted in a dry Mediterranean ecosystem focussing on *Sternbergia clusiana*, an herbaceous myrmecochore that produces an exceptionally large elaiosome and reaches its range margins within the study region. We first compared the elaiosome traits of the focal species with those of other co-occurring myrmecochorous within six sites and tested whether plants investment in reward (elaiosome) is positively correlated with the probability of interacting with the more effective ant partners. Furthermore, we tested whether such a partner choice is consistent along the geographic gradient, or whether it weakens towards the distribution margin of the focal species.

**Table 1.** Descriptions of the sites for the cafeteria experiments. Annual rainfall is calculated as the average of annual rainfall between 1981 and 2010; Average temperature is calculated as the average of January and July 1995–2009.

Site (code)	Location (long.– lat.)	Altitude (metres A.S.L)	Annual rainfall (mm)	Average temp. (°C)	Lithology	Distance from southern population (km)	Habitat	Ant species that interact with myrmecochorous seeds	Myrmechochorous species
Boker Ridge (B)	34°46′15″E 30°54′59″N	490	93	9.9–26.2	Turonian limestone	51	North-facing rocky outcrops. Vegetation is Desert Batah dominated by *Artemisia sieberi, Gymnocarpos decander* and *Pituranthos tortuosus*	*Cataglyphis savignyi; C. albicans; C. lividus; Messor ebeninus;Tapinoma israele; Monomorium venustum*	*Sternbergia clusiana; Volutaria crupinoides; Carduus getulus*
Yerucham (YR)	34°53′16″E 30°58′27″N	530	98	9.7–26.2	Cenomanian limestone	61	North-facing rocky outcrops. Vegetation is Desert Batah dominated by *Artemisia sieberi, Gymnocarpos decander* and *Pituranthos tortuosus*	*Cataglyphis albicans; C. lividus; Messor ebeninus; Tapinoma israele; Monomorium venustum*	*Sternbergia clusiana; Euphorbia hierosolymitana; Carduus getulus*
Lehavim (L)	34°49′42″E 31°22′04″N	350	297	12.1–27.8	Eocene chalk and Quaternary Loess	86	Various aspects on hills with gentle slope. Vegetation is mainly a low batah dominated by *Sacropoterium spinosum*	*Cataglyphis savignyi; C. lividus; Messor semirufus; M. ebeninus; M. arenarius; Tapinoma israele; Monomorium venustum*	*Sternbergia clusiana; Anchusa strigosa; Silybum marianum; Euphorbia hierosolymitana; Carduus getulus*
Yatir (YT)	35°02′58″E 31°21′17″N	650	271	9.7–25.3	Turonian chalk and limestone	93	Various aspects on hills with gentle slope. Vegetation is mainly a low batah dominated by *Sacropoterium spinosum*	*Cataglyphis savignyi; C. lividus; Messor semirufus; M. ebeninus; Tapinoma israele; Monomorium venustum*	*Sternbergia clusiana; Anchusa strigosa; Silybum marianum; Euphorbia hierosolymitana*
Rosh Pina (RP)	35°31′12″E 32°58′31″N	750	671	8.1–25.9	Eocene chalk and limestone	285	Steep South-facing slopes. Vegetation is a Batah dominated by *Salvia dominica*, *Majorana syriaca* and *Ononis natrix*	*Cataglyphis israelensis; Messor semirufus; Messor ebeninus; Tapinoma israele; Monomorium venustum; Crematogaster jehovae*	*Sternbergia clusiana; Silybum marianum; Euphorbia hierosolymitana; Carduus argentatus*
Mahanayim (M)	35°32′01″E 32°59′25″N	550	671	8.9–25.3	Eocene chalk and limestone	287	Steep south-facing slopes. Vegetation is a Batah dominated by *Ballota undulata, Salvia dominica*, adjacent to *Pinus haleppensis* plantation	*Cataglyphis israelensis; Messor semirufus; Messor ebeninus; Tapinoma israele; Monomorium venustum; Crematogaster jehovae*	*Sternbergia clusiana; Silybum marianum; Euphorbia hierosolymitana; Carduus argentatus*

The generality and robustness of the partner choice hypothesis can be evaluated by testing the extent to which traits, behaviours and interactions that underlie this hypothesis hold across time, space and in various contexts. Both granivorous ants and large scavenging ants are common and widely distributed in dry and open Mediterranean ecosystems (Espadaler and Gomez 1996). In those studies that focussed on myrmecochory in Mediterranean ecosystems, it was found that granivorous ant species tend to remove diaspores of many species regardless of the existence or size of an elaiosome (Garrido *et al.* 2002; Bas *et al.* 2009; Narbona *et al.* 2016). In contrast, scavenging ants do respond to the presence and size of the elaiosome (e.g. Bas *et al.* 2007; Boulay *et al.* 2007*a*). Thus, we predict that the production of elaiosomes by plants will contribute to the probability of the seeds to be dispersed by the presumably effective seed-dispersing scavenging ants. Moreover, since our study is extended from a Mediterranean into a desert biome, it also marks the distribution edge of *S. clusiana*, the myrmecochore with the largest investment in elaiosome in the study region. The unique location allows us to study partner choice towards range margins where the availability of suitable habitats for either or both partners may become more restricted and thus the spatial co-occurrence of potential partners becomes rare.

We explored variation in reward investment using the elaiosome-bearing seeds of *S. clusiana* and co-occurring myrmecochore plant species. More specifically, we investigated a number of *S. clusiana* populations across its geographical range to determine the differences in the interaction of seeds with the two co-occurring ant guilds that serve as potential dispersers with low- and high-quality dispersal services. Specifically, we addressed the following questions: (i) Do co-occurring myrmecochore species exhibit variation in reward investment and, in particular, in elaiosome mass and in elaiosome to seed mass ratio?; (ii) Is the investment in reward correlated with higher dispersal quality? and (iii) If indeed there is a relationship between reward investment and dispersal quality (as reflected by interaction intensities with the two ant guilds), is it consistent along a geographic gradient? We hypothesized that the seeds of species that invest more in the elaiosome will be consistently removed (a) at higher rates, (b) more so by scavenging ants and (c) that the effect of reward size on seed removal rate will decrease towards the species range limit. This last prediction stems from the distribution of suitable habitat for *S. clusiana* in the southern range limit, which consists of small and isolated patches (Boeken and Guttermann 1989). Such a distribution fits very well a scenario at non-expanding range limit, which selects for limited dispersal (Dytham 2009; Hargreaves *et al.* 2015) and thus low investment in reward.

## Materials and Methods

### Study species and study sites

The study was conducted along a steep geographic gradient in the eastern Mediterranean region. This region has a very rich flora, with a modest representation of myrmecochors and a dearth of myrmecochory studies (Warren and Giladi 2014). The focal plant species of our investigation is *Sternbergia clusiana* (Amaryllidaceae), a myrmecochore bulbous herbaceous perennial. It exhibits unique phenology, with flowers emerging from the exposed soil in September–December following a long and dry summer. The leaves emerge in December–March with the onset of the rains, and the fruits mature and then release the seeds in March–April, 5 months after flowering (Boeken and Guttermann 1989). The fruits are oval, 1–3 cm long and ~0.7 cm in diameter. Fruits contain highly variable numbers $\left(27.6\pm 20.85;\;\bar{X}\pm SD\right)$ of diaspores (seed + elaiosome). The seed is among the heaviest of any myrmecochore species in the Mediterranean region (109.58 ± 40.49 mg).

**Fig. 1.**
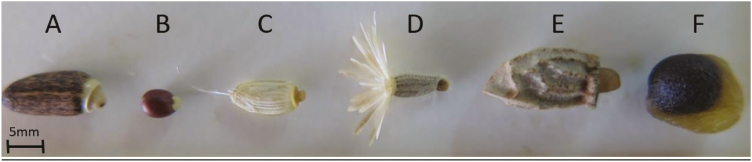
Photographs of diaspores used for cafeteria experiments: (A) *Silybum marianum*, (B) *Euphorbia hierosolymitana*, (C) *Carduus getulus/C. argentatus*, (D) *Volutaria crupinoides*, (E) *Anchusa strigosa* and (F) *Sternbergia clusiana*.

The species distribution ranges from Eastern Turkey and Western Iran in the north to Jordan and Israel in the south (Mathew 1984; Youssef *et al.* 2017). Within our study region, the species is found in about ~60 sites distributed along a steep precipitation gradient ranging from Mediterranean climate in the north (>600 mm annually) to arid climate in the Negev highlands (<100 mm), where *S. clusiana* reaches its southern range limit. The populations within the vast majority of these sites are locally confined to a specific well-defined habitat type and are of limited extent, thus forming clearly isolated populations. Within this gradient, we chose six *S. clusiana* populations representing the geographical distribution of the species towards its southernmost distribution edge (Table 1). Within each of the selected sites, we recorded all the co-occurring myrmecochore species (Table 2). Out of the six additional myrmecochore species found, one was common to all sites, while the others had a more restricted distribution. Out of the various diaspore characteristics of the myrmecochore plant species (Fig. 1), we focussed on the investment in the elaiosome, the reward that is likely to alter ant behaviour.

**Table 2.** Co-occurring myrmecochore species. Distribution represents the study sites in which the species were observed (see Table 1 for site codes).

Species name	Family	Life form	Diaspore description	Distribution
*Silybum marianum*	Asteraceae	Therophyte	Diplochorous (anemochory and myrmecochory) with a mid-size elaiosome; located at the top of the seed; easily detachable pappus	L, YT, M, RP
*Euphorbia hierosolymitana*	Euphorbiaceae	Chamaephyte	Diplochorous (ballistic and myrmecochory); Elaiosome is relatively small	B, L, YT, M, RP
*Carduus getulus*	Asteraceae	Therophyte	Diplochorous (anemochory and myrmecochory) with a small-size elaiosome elaiosome located at the base of the style; easily detachable pappus	B, L, YT
*Carduus argentatus*	Asteraceae	Therophyte	Diplochorous (anemochory and myrmecochory) with a small-size elaiosome located at the base of the style; easily detachable pappus	M, RP
*Volutaria crupinoides*	Asteraceae	Therophyte	Diplochorous (anemochory (?) and myrmecochory) with a small-size elaiosome located at the top of the style; stiff pappus	B
*Anchusa strigosa*	Boraginaceae	Hemicryptophyte	Myrmecochore; large non-juicy elaiosome	L, YT

Harvester ants (mainly of the genus *Messor*) and large scavenging ants (mainly of the genus *Cataglyphis*) are very common throughout the region. In preliminary observation, we found that the ant species that are most likely to interact with seeds of myrmecochores are large granviorous of the *Messor* genus (*M. ebeninus, M. arenarius* and *M. semirufus*) and medium to large scavenging ants of the *Cataglyphis* genus (*C. israelensis*, *C. savignyi* and *C. albicans*). In addition, some smaller ant species, mainly *Tapinoma israelensis* and *Mononorium* sp. occur in some of the study sites, approach seeds, occasionally gnaw the elaiosome on the spot, but are too small to actually remove the seed.

### Diaspore collection and evaluation of investment in reward

During March–May 2016 we visited each site on a weekly basis to examine whether the fruits of any of the local myrmecochore species have ripened. At time of fruits ripening, diaspores were collected from at least 10 individuals of each plant species. For the determination of seed and diaspore masses, we used five diaspores from each of 10 individuals per site. For *S. clusiana,* the wet mass of 100 diaspores (5 from each of 20 individuals) was weighed. In all species, elaiosomes were removed by hand/scalpel and the intact seeds were weighed again after elaiosome removal and the mean elaiosome to seed mass ratio was calculated (Table 2). Because preliminary experiments showed that ants of both guilds do not discriminate between freshly collected diaspores and diaspores that were stored in advance, we stored diaspores that were not used for the mass measurement at −18 °C and used them in the cafeteria experiments.

### *In situ* cafeteria experiments

We conducted a set of *in situ* cafeteria experiments to test whether elaiosome traits affect diaspore choice by different seed-dispersing ant guilds. A depot was composed of simultaneous presentation of five diaspores of each local myrmecochore species in addition to a control, composed of five seeds of quinoa (*Chenopodium quinoa*; that does not contain an elaiosome). In each of the six study sites, we located 10 active colonies of each of the ant guilds and simultaneously presented each colony with two seed depots. One depot was placed next to the nest entrance (0 m) and another at a distance of 1–1.5 m from the nest opening, where active foragers were observed. Experiments were conducted between 15 April and 15 May 2016 to coincide with the natural seed release time of the studied species and only at time when ant colonies exhibited high foraging activity. We monitored depots until all diaspores were removed, or up to a maximum observation time of 1 h. During the monitoring, we noted which diaspores were taken, when and by which ant species. Overall, a total of 5790 diaspores were placed at 253 seed depots during this experiment. In addition to removal rates, during the cafeteria experiments, we classified all visiting ant–seed interactions into six categories, using a behavioural index modified from Culver and Beattie (1978): (0) ignore—no sign of interaction with the diaspore; (1) unintentional contact (touch and go)—where the ant passes through the seed depot and shows no sign of interaction with the diaspore; (2) antennate—the ant touches the diaspore with her antennae; (3) examine—the ant stops for several seconds, uses her mouthparts, but does not try to pick up the diaspore; (4) pickup—the ant tries to pick up the seed, or roll it over, but moves it less than 10 cm; and (5) remove—the ant moves the seed at least 10 cm from the seed depot.

### Statistical analysis

Differences among plant species in investment in reward, measured as absolute and proportional fresh elaiosome biomass, were tested using generalized linear-mixed model (GLMM). The plant species was treated as a fixed factor and the different individuals as random factor nested within a species, using normal distribution with log-link function for the elaiosome mass and identity link function for the elaiosome/diaspore mass ratio. These were followed by a Tukey test.

In order to test whether the two ant guilds interact differently with the various myrmecochore species and, in particular, differ in their tendency for diaspore removal, the rate of diaspore removal was analysed using two complementary approaches: first, to asses diaspore removal dynamics in the cafeteria experiments, we conducted survival analyses (=proportion of diaspore left) of each myrmecochore species per site in relation to the effect of ant guild using log-rank test (Kleinbaum and Klein 2012). Second, we used generalized linear models (GLMs) in which we tested for the probability of a diaspore to be removed from a depot by the end of experimental trial using a binomial distribution and a logit-link function. We conducted three sets of analyses: to test whether the two ant guilds differ in their overall seed dispersal activity (regardless of seed identity) towards the range margin, we tested for the effect of ant guild, site and their interactions on the probability of seed removal. Then, in order to test whether ant guilds differ in their preference of diaspore species (regardless of site), we tested for the effect of ant guild, diaspore species and their interaction on the probability of seed removal. Following these analyses, and because not all plant species were present at all the six study sites, we also analysed the effect of ant guild, diaspore species and their interactions for each site separately. In addition, in order to test differences in the nature of the behavioural interaction index between the two ant guilds and the different sites, we used a GLM. Here we tested the effects of site, ant guild and their interaction using normal distribution with identity link function. All analyses were conducted with SPSS 24.0 (IBM Corp. Released 2013. IBM SPSS Statistics for Windows, Version 24.0. Armonk, NY: IBM Corp.).

## Results

### Elaiosome traits

**Fig. 2.**
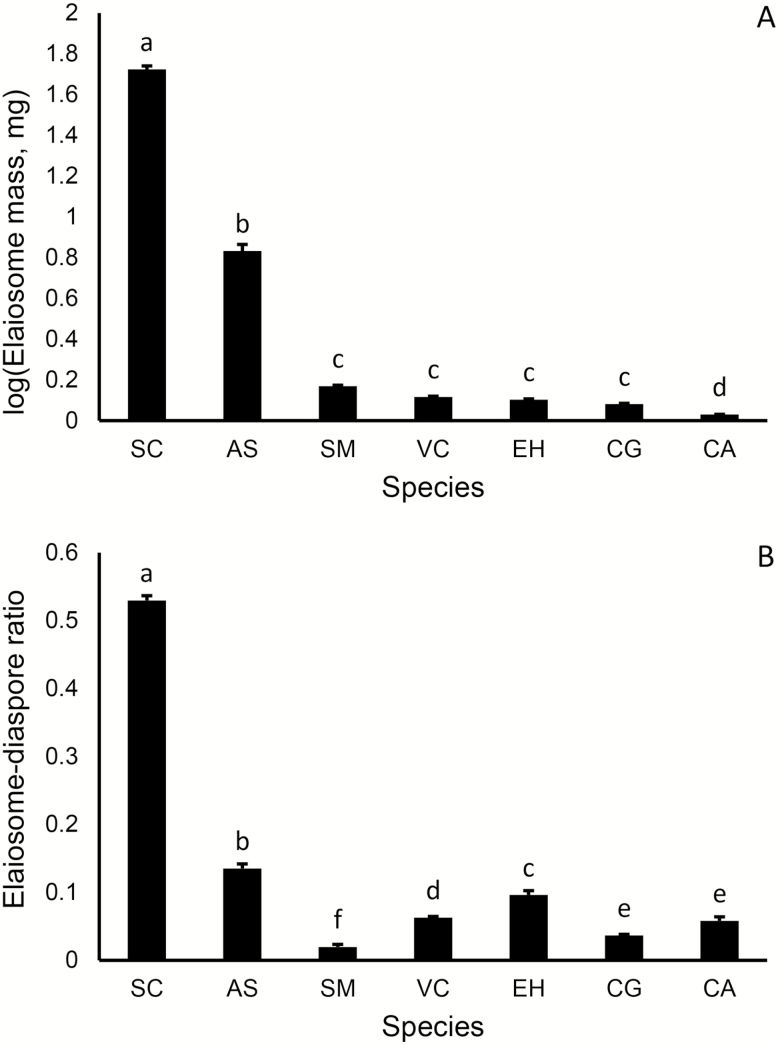
Interspecies variation in diaspore traits: (A) Mean (±SE) elaiosome biomass of the co-occurring myrmecochore species, data are log-transformed. (B) Mean (±SE) elaiosome/diaspore ratios of the co-occur myrmecochore species. Different letters indicate significant (*P* < 0.05) differences between species as evaluated by pairwise comparisons. SC, *Sternbergia clusiana*; AS, *Anchusa strigosa*; SM, *Silybum marianum*; VC, *Volutaria crupinoides*; CA, *Carduus argentatus*; CG, *Carduus getulus*; EH, *Euphorbia hierosolymitana*; CQ, *Chenopodium quinoa*.

Differences in elaiosome mass between the different myrmecochore species were significant (*F*_6,71_ = 695.9, *P* < 0.001, see Fig. 2A), as well as those between elaiosome/diaspore mass ratio (*F*_6,71_ = 313.9, *P* < 0.001, see Fig. 2B). The ranking of species by absolute elaiosome mass was similar to the ranking by elaiosome/diaspore mass ratio, with *S. clusiana* exhibiting the highest investment in ant reward, followed by *A. strigosa.* The other myrmecochore species exhibited much lower absolute and relative investment in the elaiosome.

### *In situ* cafeteria experiments

**Fig. 3.**
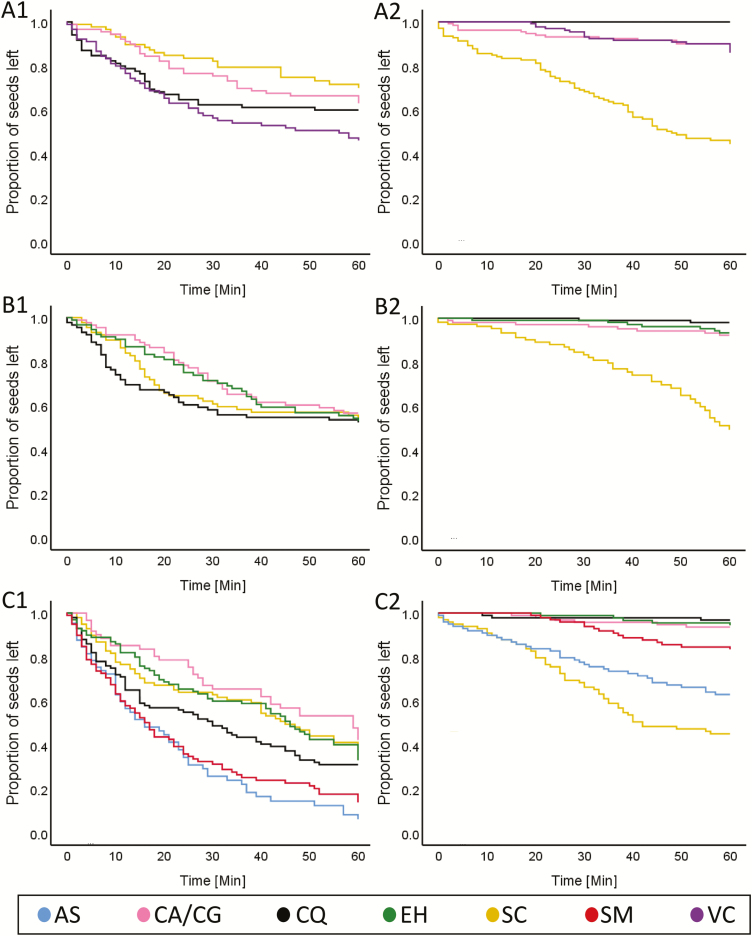

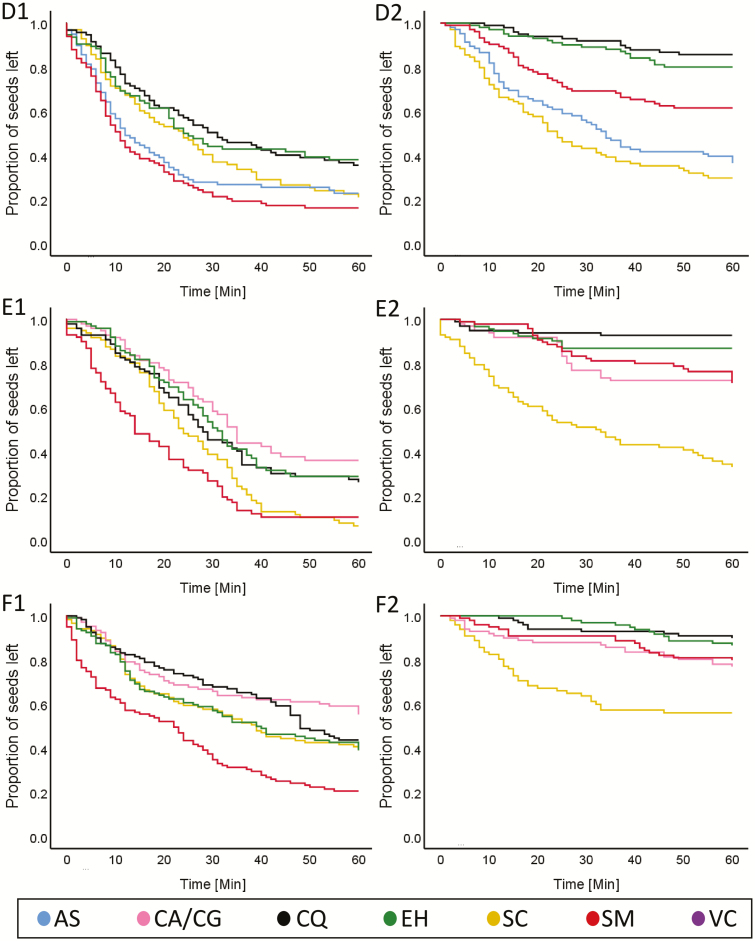
Removal probability (based on survival analysis) of a diaspores by Granivorous (left) and scavenging ants (right) as a function of diaspore species at Boker (A), Yerucham (B), Lehavim (C), Yatir (D), Rosh Pina (E) and Mahanayim (F) sites. Diaspore species: SC, *Sternbergia clusiana*; AS, *Anchusa strigosa*; SM, *Silybum marianum*; VC, *Volutaria crupinoides*; CA/CG, *Carduus argentatus/C. getulus*; EH, *Euphorbia hierosolymitana*; CQ, *Chenopodium quinoa*.

We observed more than 3000 diaspore–ant interactions. Most of these interactions (93 %) involved six ant species from two genera: three species of the granivorous *Messor* genus (*M. ebeninus, M. arenarius* and *M. semirufus*) and three species of the scavenging *Cataglyphis* genus (*C. israelensis*, *C. savignyi* and *C. albicans*). Removal of diaspores by foraging ants (regardless of the identity of the myrmecochorous species) accounted for 41 % of the ant–seed interactions. When analysing the differences between the two ant guilds in the dynamics of diaspore removal using survival analysis, we found that overall seed removal rates were similar among the six study sites: granivorous ants tended to remove all diaspores at a steady rate during the 60 min of observations. Scavenging ants showed a tendency to remove large elaiosome diaspores (especially those of *S. clusiana*) faster than smaller diaspores. Furthermore, this trend of preference for one diaspore over the others was often observed from the onset of the 60 min cafeteria experiments (Table 3; Fig. 3).

**Table 3.** Log-rank test of equality of survival distributions for the different diaspore species. Bold: *P*-value <0.001; *: 0.01 < *P*-value < 0.05.

Site	Granivore	Scavenger
	$\chi^{2}$(df)	$\chi^{2}$(df)
Boker	10.70(3)*	**182.17(3)**
Yerucham	1.20(3)	**118.24(4)**
Lehavim	**55.45(5)**	**155.54(5)**
Yatir	**29.00(4)**	**136.36(4)**
Rosh Pina	**46.61(4)**	**69.55(4)**
Mahanayim	**43.73(4)**	**53.74(4)**

**Fig. 4.**
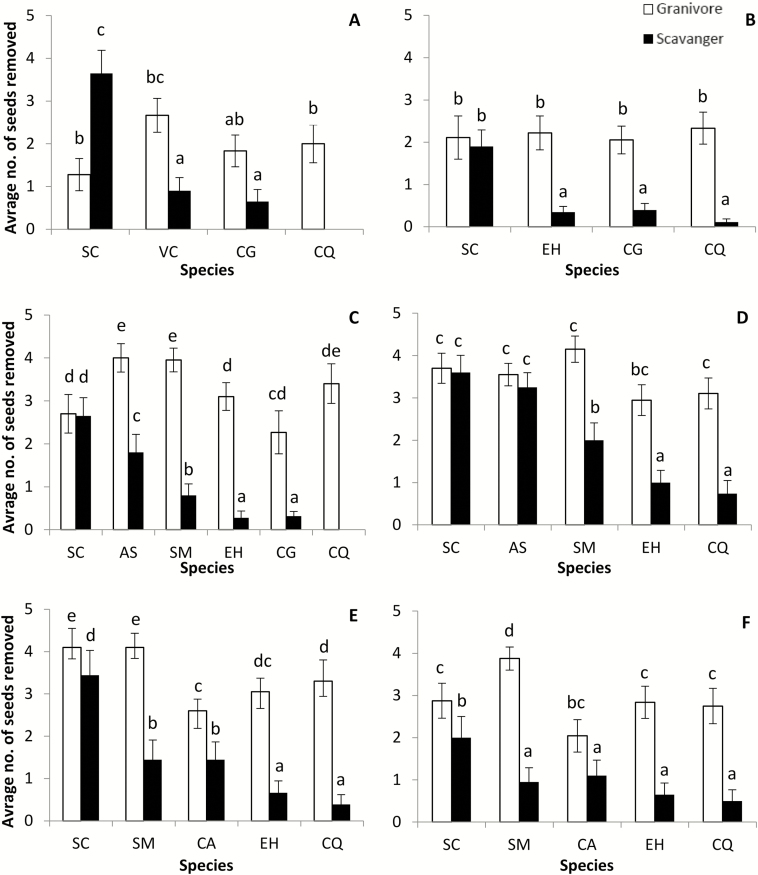
Average removal rates of diaspores by scavenging ants (black bars) and granivorous ants (empty bars) for the co-occurring species at the six sites. (sites: A, Boker; B, Yerucham; C, Lehavim; D, Yatir; E, Rosh Pina; F, Machanayim.) Plant species: SC, *Sternbergia clusiana*; AS, *Anchusa strigosa*; SM, *Silybum marianum*; VC, *Volutaria crupinoides*; CA, *Carduus argentatus*; CG, *Carduus getulus*; EH, *Euphorbia hierosolymitana*; CQ, *Chenopodium quinoa*. Species are ordered in descending order according to their elaiosome mass (see Fig. 1A). Different letters indicate significant (*P* < 0.05) as evaluated by pairwise comparisons.

Overall, the probability of a diaspore removal by a granivorous ant (regardless of diaspore species) was significantly higher than that of a diaspore being removed by a scavenging ant (ant guild *χ*^2^_1_ = 663.70, *P* < 0.001). The overall diaspore removal probabilities were lower in the southern sites, which are the most arid and the closest sites to the range margin of *S. clusiana* (site χ^2^_5_ = 216.62, *P* < 0.001). Moreover, we detected a significant interaction between site and ant guild in their effect on overall diaspore removal (ant guild × site χ^2^_5_ = 32.66, *P* < 0.001, Fig. 4). When testing the effect of ant guild and diaspore species on removal probabilities (regardless of site), we found that diaspore removal was significantly different among diaspore species (diaspore species, χ^2^_7_ = 426.60, *P* < 0.001). Moreover, the probability of seeds to be removed by granivorous ants was higher than that of removal by scavenging ant (ant guild *χ*^2^_1_ = 541.87, *P* < 0.001). The effect of ant guild on removal differed between diaspore species (ant guild × diaspore species χ^2^_7_ = 289.27, *P* < 0.001), indicating differences between the ant guilds in preference of seed species. In the third set of analyses of diaspore removal, when preferences of seeds by the two ant guilds were tested separately for each site (Table 4; **see Supporting Information—****Table S1**), we found that in all sites, scavenging ants removed the diaspores of species with larger elaiosome at higher rates than they removed small elaiosome species (Fig. 4). Unlike the scavenging ants, granivorous ants showed little preferences in their diaspore choice, and no consistent association was detected between elaiosome/seed ratio and removal probability. Moreover, while the diaspores of the non-myrmecochore Quinoa were removed at high rates by granivorous ants, they were almost completely ignored by the scavenging ants.

**Table 4.** Generalized linear model, binomial distribution (Logit function) results for diaspore removal as a function of ant guild, plant species and the interaction between plant species and ant guild. Diaspore removal data are split by sites. Bold: *P*-value <0.001.

Site	Ant guild	Plant species	Plant species × ant guild
	Wald $\chi^{2}$ (df)	Wald $\chi^{2}$ (df)	Wald $\chi^{2}$ (df)
Boker	**1078.65 (1)**	**2242.29 (3)**	**3678.81 (2)**
Yerucham	**72.38 (1)**	**2222.06 (4)**	**34.12 (4)**
Lehavim	**195.57 (1)**	**68.21 (5)**	**77.95 (5)**
Yatir	**81.76 (1)**	**3757.16 (5)**	**39.96 (5)**
Rosh Pina	**150.63 (1)**	**89.87 (4)**	**30.78 (4)**
Mahanayim	**148.95 (1)**	**28.37 (4)**	**31.82 (4)**

**Fig. 5.**
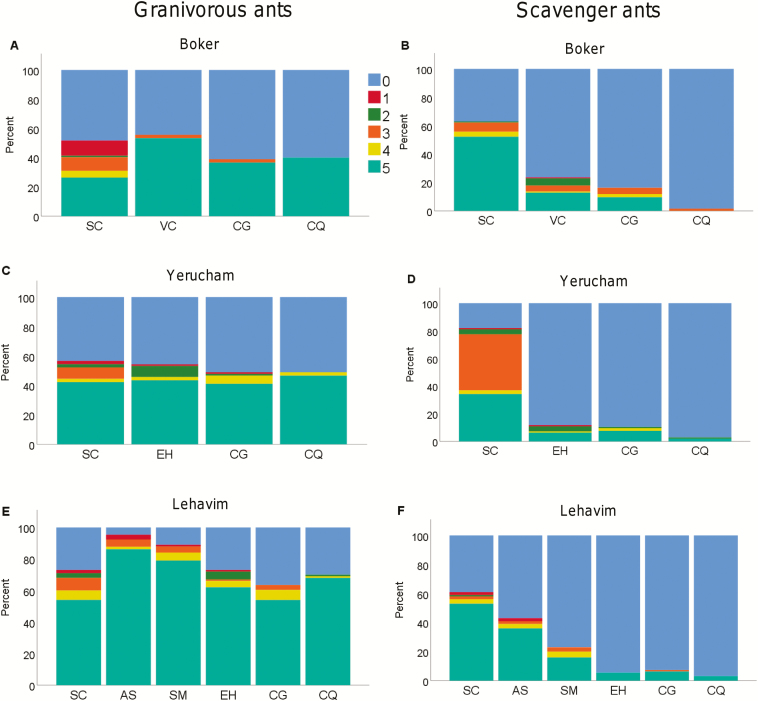

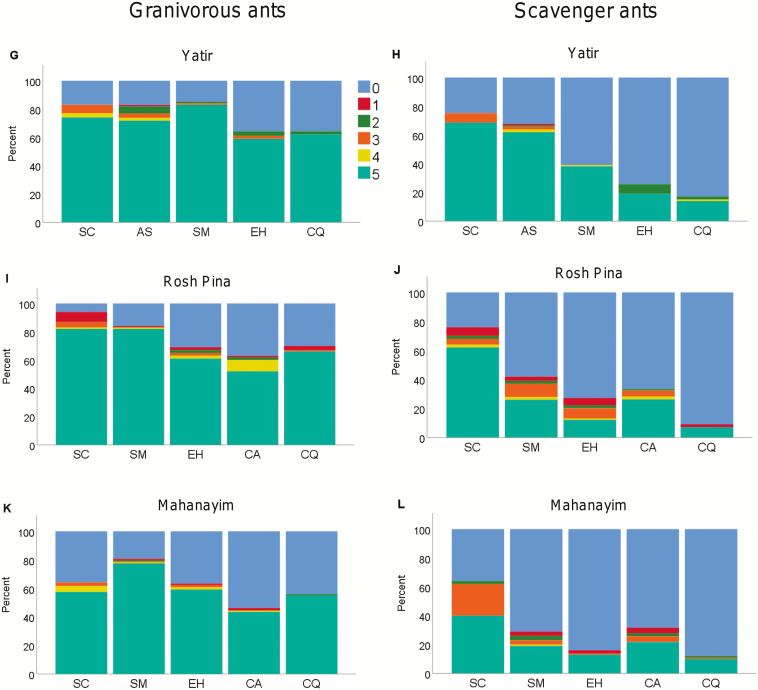
Relative proportion of behavioural interaction indices between diaspores of the plant species and the ant guilds. Left column is interactions by granivorous ants, and right is interactions by scavenging ants. (0) blue—‘Ignore’: no sign of interaction with the diaspore. (1) Red—unintentional contact ‘touch and go’—where the ant passes through the seed depot and shows no sign of interaction with the diaspore. (2) Green—‘Antennate’: the ant touches the diaspore with her antennae. (3) Orange—‘Examine’: the ant stops several seconds, uses her mouthparts, but does not try to pick up the diaspore. (4) Yellow—‘Pick-up attempt’: the ant tries to pick up the diaspore, or roll it over, but moves it less than 10 cm. (5) Light blue—‘Removal’: the ant moves the diaspore at least 10 cm. Different letters indicate the study sites: A, Boker; B,Yerucham; C, Lehavim; D, Yatir; E, Rosh Pina; F, Machanayim.

The most common behavioural interactions between ants of both guilds and the diaspores were the two extreme: ‘ignore’ (0) or ‘remove’ (5). Overall, the average intensity of the behavioural interaction index was higher for granivorous ants relative to scavenging ants (ant guild *χ*^2^_1_ = 40.26, *P* < 0.001) and decreased towards the southern sites (site χ^2^_5_ = 72.78, *P* < 0.001). In addition, a significant interaction between site and ant guild was detected (ant guild × site χ^2^_5_ = 26.24 *P* < 0.001): for the granivorous ants, ‘remove’ and ‘ignore’ types were at similar frequencies in the two southern sites, but diaspore ‘remove’ was more common than ‘ignore’ in the other four sites (Fig. 5). In contrast, for scavenging ants, ‘remove’ frequencies were higher and ‘ignore’ was lower when interacting with *S. clusiana* seeds and the balance shifted to mainly ‘ignore’ and less ‘remove’ for seeds with lower elaiosome/seed ratio (Fig. 5). There was one exception to this general pattern in one site (Yerucham) (Fig. 5B), where scavenging ants were equally likely to just examine seeds of *S. clusiana* seeds as to remove them.

## Discussion

Our main goal was to test whether the main features that characterize partner choice—variation in investment by plants, differential response by different ant guilds and the match between plant traits and ant response—are consistent across a geographic gradient. We utilized the extent of variation in reward investment by co-occurring plant species to test its potential impact on variation in dispersal quality, as reflected by the frequency of interaction and the foraging behaviour of the two ant guilds.

Our study supports the generality of the partner choice hypothesis with two main findings. First, we showed that interspecies variation in elaiosome size and elaiosome/diaspore mass ratio among co-occurring species is substantial. With the possible exception of *A. strigosa*, which produces a relatively heavy elaiosome, the investment in the elaiosome as a reward by all other species was much lower than that of *S. clusiana*. Such high among-species variation in elaiosome size and elaiosome/diaspore ratio has been reported in several other ecosystems, such as in temperate forests in central Europe (Reifenrath *et al.* 2012), the Caatinga vegetation in Brazil (Leal *et al.* 2014) and Australia (Edwards *et al.* 2006).

Second, we found that scavenging ants always preferred seeds of plant species that produced large elaiosomes (e.g. *S. clusiana*). This preference was consistent along the geographic gradient, regardless of the vicinity of the site to the range margin of *S. clusiana*, and regardless of the representation of other elaiosome-producing species in the local plant community. This behaviour fits observations from other systems where scavenging ants function as keystone dispersers (Gove *et al.* 2007; Ness *et al.* 2009; Lubertazzi *et al.* 2010), and is considered as the norm in other ecosystems where this ant guild is present (Oostermeijer 1989; Hughes and Westoby 1992; Leal *et al.* 2014). In addition to the clear behavioural response to elaiosome size, scavenging ants in many other ecosystems are also numerically the most dominant guild in the removal of myrmecochores seeds (Gove *et al.* 2007; Ness *et al.* 2009). Unlike the scavenging ants, granivorous ants did not respond to the presence or size of elaiosomes, concurring with results in other studies (Perez *et al.* 2006). However, in contrast with the numerical dominance of scavenging ants in most myrmecochorus systems (Gove *et al.* 2007; Ness *et al.* 2009), in our study system granivorous ants were common at all sites and numerically as important as scavenging ants in the removal of mrymecochore’s seeds.

The large scavenging ants seemed to respond to the absolute mass of the elaiosome rather than to the elaiosome/diaspore mass ratio. Such a preference pattern is expected if the energetic gain from a food item (e.g. elaiosome) significantly outweigh the energetic cost of carrying the load of the whole diaspore (Fewell 1988; Mark and Olesen 1996; Peters *et al.* 2003; Bas *et al.* 2007). This may indeed be the case for the larger scavenging ants in our study region (*C. savignyi* and *C. israelensis*) that carry large seeds at high running velocities (N.L. and G.B.Z., personal observation), which may indicate a low relative energetic demand. This stands in contrast with finding in Australia myrmecochoreous systems, where positive allometric relationships between elaiosome and seed masses were found, and were interpreted as indicating a high energetic cost paid by scavenging ants (Edwards *et al.* (2006). Such allometry is predicted in ecosystems where legitimate dispersers are common and are the target of selection. In ecosystems where granivores are plentiful and more dominant than legitimate dispersers (scavengers), investment in elaiosome should be sufficient to reduce predation risk, but size may be less tightly selected. Thus, we may expect investment in elaiosome to be more variable, as observed in our study system. Another trait that may have affected ant preferences is that *S. clusiana* produces a fleshy elaiosome, whereas the elaiosome of the other species are much drier and even though they may retain a low attractiveness for longer periods.

Geographic heterogeneity in various myrmecochory’s features of both ants and plants has been documented in numerous studies previously (Garrido *et al.* 2002; Alcantara *et al.* 2007; Boulay *et al.* 2007*b*, Manzaneda and Rey 2008; Ehlers 2012). Some studies focussed on spatial variation in myrmecochory characteristics at a local scale and found that a-biotic conditions are limiting the activity of the more effective dispersers and consequently affected dispersal quality and the plant spatial distribution (Giladi 2004; Ness 2004; Ness and Morin 2008; Warren *et al.* 2010). Studies conducted along elevational gradient found that diaspore removal rates by ants decreased with elevation due to the change in the density and diversity of effective seed-dispersing ants, which in turn correlate with gradient-related climatic variables (Zelikova *et al.* 2008; Thomson *et al.* 2016). Similarly, a study that spans a wide geographic range showed that removal rates of a European myrmecochore (*Helleborus foetidus*) are much affected by the species and the functional group composition of the local ant community (Manzaneda and Rey 2008). However, most of these studies of myrmecochory along geographic gradients include or approach the range limit of a single myrmecochore. Our study is unique in that we simultaneously examined the interaction between two main ant guilds and the local myrmecochore community in each site along such gradient.

The simultaneous presentation of seeds of different plant species and the pairing of observations of the two ant guilds in the cafeteria experiments allowed us to focus on the interaction of plants investment and the ant guilds. Ideally, we would conduct the cafeteria experiments with the same set of ant species throughout the gradient. However, not all ant species were present in all sites (Table 1). Instead, as representative of each guild we chose species that are ecologically equivalent and focussed on guild-level understanding. Yet, the observed differences among the sites in overall seed removal rates and in the relative contribution of each ant guild could, at least partially, be due to differences in the species representing each ant guild within each site. For example, in independent observations conducted in the same six sites, we found that the overall activity level of scavenging ants, as measured by visits to baits, was higher in Lehavim and Yatir than in all other sites. In the two southern sites, large scavenging ants (e.g. *C. savignyi*) were either absent (Yeruham) or locally restricted (Boker), so that scavenging ants in these sites were mainly represented by the smaller species, C. *albicans*. Although *C. albicans* occasionally attempted to remove seeds of *S. clusiana*, they mostly failed and more frequently just examined the seeds. In general, it is assumed that small ant species provide lower quality dispersal services than large ant species (Ness *et al.* 2004; Manzaneda *et al.* 2007; Leal *et al.* 2014), or may even harm dispersal by consuming the elaiosome on spot (Bond and Breytenbach 1985; Oostermeijer 1989; Manzaneda and Rey 2009; Rowles and O’Dowd 2009).

Geographic variation in seed dispersal may be due to both among-guilds composition and within-guild composition of the ant community (e.g. Manzaneda and Rey 2008). In the case of the granivorous ant community, in the southern sites, *M. ebeninus* is the most abundant, *M. arenarius* is scarce and *M. semirufus* is absent. In the central sites, all three species are equally distributed, and in the northern sites we found only *M. semirufus* nests. These species differ in their body size, where *M. ebeninus* workers are the smallest, *M. semirufus* workers are intermediate and *M. arenarius* workers are the largest (Segev *et al.* 2014). Since there is a positive correlation between ant body size the size of seeds that it can carry (see Ness *et al.* 2004), we suggest that ant body size is an important source of variation in mean removal rates, removal dynamic and interaction frequency by granivorous ants across the geographic gradient.

Investment in large elaiosome is likely to benefit a plant as it increases the frequency of interacting with the larger scavenging ants. However, this is not direct evidence that these ants are high-quality dispersers. Previous studies have found that reward size is not only positively correlated with higher attractiveness to large scavenging ants, but also results in higher dispersal quality (Ohara and Higashi 1987; Hughes and Westoby 1992; Mark and Olesen 1996; Bas *et al.* 2007; Ehlers 2012; Tanaka and Tokuda 2016; Zhu *et al.* 2017; but see Gunther and Lanza 1989; Imbert 2006; Reifenrath *et al.* 2012; Turner and Frederickson 2013). This high-quality dispersal has been associated with specific characteristics of keystone dispersers (e.g. *Aphaenogatser* in North America, Ness *et al.* (2009), *Rhytidiponera* in southwestern Australia (Gove *et al.* 2007)). Those keystone dispersers do not consume seeds (only the elaiosome), keep small colony size, frequently relocate their nest, forage solitarily, have excellent food detection capabilities and redisperse seeds beyond the nest limit (see Warren and Giladi (2014) for a detailed list). The large *Cataglyphis* species in our system (mainly *C. savignyi* and *C. israelensis*) share many of the traits characterizing well-known keystone dispersers (Gorb and Gorb 1995; Boulay *et al.* 2007*b*; Gove *et al.* 2007; Barroso *et al.* 2013; Leal *et al.* 2014). Furthermore, the results from this study and our additional observations indicate that large *Cataglyphis* in our system disperse myrmecochorous seeds at high rates, to long distances (up to 40 m) and redisperse the seeds to favourite sites for plants establishment.

Although seed removal rates by granivorous ants in our system were high, the effectiveness of such removal for seed dispersal is more questionable. This is mainly due to the dual role that granivorous ants usually play as both seed predators and seed dispersers (Espadaler and Gomez 1996; Gomez and Espadaler 1998; Boulay *et al.* 2009; Warren and Giladi 2014), with the net outcome shifting from negative to positive in relation to plant traits, ant traits and the environment (Arnan *et al.* 2012). Overall, diaspore removal rates by granivorous ants, as measured in our cafeteria experiments, were similar or even higher than those by scavenging ants (see Fig. 3). However, while these results clearly represent removal tendencies by the different guilds once diaspores are encountered by the ants, their interpretation should be taken with a grain of salt. In the case of the scavening ants, we placed the experimental seed deopts at random locations around the nest opening. However, due to the poor ability of the granivorous ants in our system to quickly detect new food resources (Avgar *et al.* 2008), we placed experimental seed depots along active foraging trails. This design served well our goal to test for preferences by ants of diaspores with various rewards level. However, it introduced a bias in favour of granivorus ants when it comes to estimating overall removal rates. Thus, the removal rates that we measured for granivorus ants, probably overestimated the contribution of this guild of ants to overall seed removal rates under natural distirbution of seeds.

Finally, we argue that the functionality of exceptionally small elaiosome, as produced by some plant species in our system, in attracting efficient seed-dispersing ants should be carefully scrutinized. Five of the seven plant species included in this study are diplochorous, where myrmecochory serves as a secondary dispersal mechanism. Four species (the two *Carduus* spp., *S. marianum* and *V. crupinoides*) express adaptations for wind dispersal, whereas *E. hierosolymitana* has a ballistic dispersal as a primary dispersal mechanism. Previous studies suggest a tradeoff exists between plant’s investments in the two mechanisms, which results in diplochorous species having smaller elaiosomes than ‘pure’ myrmecochores (Ohkawara and Higashi 1994; Narbona *et al.* 2005; Narbona *et al.* 2016). Our results agree with the existence of such tradeoff. Moreover, given the low removal rates of diplochorous diaspores by the scavenging ants in our study, the potential advantage that elaiosomes may provide to these species should be questioned.

In conclusion, this study demonstrates that both the variation in plant traits and the corresponding response of different ant guilds, which are necessary for a partner choice to occur, are consistent along a sharp geographic gradient. This consistency holds even in the presence and dominance of apparently low-quality dispersers, which remove seeds regardless of the plant investment. This may indicate that the selection by scavenging ants is sufficiently strong to justify the investment in reward even when low-quality partners are common. Alternatively, the investment in reward observed in our system may result from and reflect selection pressures that operated elsewhere and/or at other times, but are fixed at the species level (Thompson 1999). A study that will focus on spatial heterogeneity in the functional benefits of myrmecochory along the gradient will be needed to tease apart these explanations.

## Availability of Data and Material

The data used for the analyses are also available as Supplementary Information.

## Conflict of Interest

None declared.

## Contributions by the Authors

All authors conceived and designed the study. N.L. and G.B.Z. collected the data. N.L. wrote the first draft, conducted the statistical analysis and led the revisions with major contributions by M.S. and I.G.

## Sources of Funding

This work was supported by graduate student Fellowship from the Blaustein Institutes for Desert Research to N.L. This study was supported by an Israel Science Foundation grant (No. 834/15) to I.G.

## Supplementary Material

plz027_suppl_Supplementary-Data-1

plz027_suppl_Supplementary-Data-2

plz027_suppl_Supplementary-Table-A1

## Supplementary Information

The following additional information is available in the online version of this article—

Table S1: Coefficients (SE) for diaspore removal as a function of ant guild, plant species and their interaction.
