# Supplementary material for: Investment in reward by ant-dispersed plants consistently selects for better partners along a geographic gradient
Source: AoB Plants. 2019 May 2;11(3):plz027. doi: 10.1093/aobpla/plz027 (PMC6534284; doi:10.1093/aobpla/plz027)
Supplement: plz027_suppl_Supplementary-Table-A1 [file plz027_suppl_supplementary-table-a1.pdf]

Table A1

Coefficients (SE) for diaspore removal as a function of ant guild, plant species and their interaction. Models were performed for each site separately as shown in table 4. Site abbreviations: B – Boker; YR – Yerucham; L – Lehavim; YT – Yatir; RP – Rosh Pina; M – Mahanaim. Plant species abbreviations: SC - *Sternbergia clusiana*; AS – *Anchusa strigose*; SM – *Silybum marianum;* VC – *Volutaria crupinoides;*EH – *Euphorbia hierosolymitana;* CG – *Carduus getulus;* CA – *Carduus argentatus*; CQ – *Chenopodium quinoa*. Bold values P<0.001; ** 0.001<P<0.01; * 0.01<P<0.05.

	B	YR	L	YT	RP	M
Granivores (G)	**2.06(0.36)**	0.23(0.30)	**2.98(0.37)**	**2.07(0.33)**	**2.47(0.35)**	**2.69(0.25)**
SC	**2.29(0.35)**	-0.55(0.21)**	**1.78(0.34)**	**1.27(0.29)**	**1.70(0.33)**	1.05(0.33)**
AS			1.08(0.34)**	**1.21(0.29)**		
SM			**-1.67(0.27)**	-0.49(0.20)*	**-0.96(0.23)**	**-1.45(0.25)**
VC	**-1.93(0.29)**					
EH		**-2.14(0.44)**	-1.18(0.53)*	-0.96(0.32)**	-1.03(0.39)**	-0.45(0.38)
CG	-0.67(0.47)	**-1.95(0.42)**	-1.04(0.50)*			
CA				**-3.03(0.20)**	-0.09(3.3)	0.14(0.35)
CQ	**-2.78(0.31)**	**-3.38(0.74**)	-1.77(0.65)*	**-1.33(0.35)**	**-1.62(0.46)**	-0.75(0.42)
G×SC	**-3.50(0.47)**		**-2.94(0.46)**	**-1.81(0.46)**	-1.70(0.49)*	**-1.98(0.43)**
G×AS			-0.58(0.55)	**-1.90(0.46)**		
G×EH		**2.19(0.53)**	0.34(0.62)	-0.26(0.47)	-0.44(0.51)	-0.42(0.48)
G×CG	-0.01(0.56)	**1.90(0.52)**	-0.13(0.61)			
G×CA					-1.35(0.46)*	**-1.64(0.46)**
G×CQ	**27.30(0.28)**	**3.56(0.80)**	1.19(0.72)	0.24(0.49)	0.77(0.57)	-0.29(0.51)
